# Supplementary material for: Yiai Fuzheng decoction inhibits triple-negative breast cancer by remodeling the immune microenvironment
Source: Front Immunol. 2025 Sep 30;16:1615631. doi: 10.3389/fimmu.2025.1615631 (PMC12518410; doi:10.3389/fimmu.2025.1615631)
Supplement: Supplementary file 2 [file DataSheet1.zip › Supplementary File 2/Treatment/D_vs_H/07_Pathway_Analysis/Enrichment_Analysis/smpdb_pathway/Enrichment_Barplot.pdf]

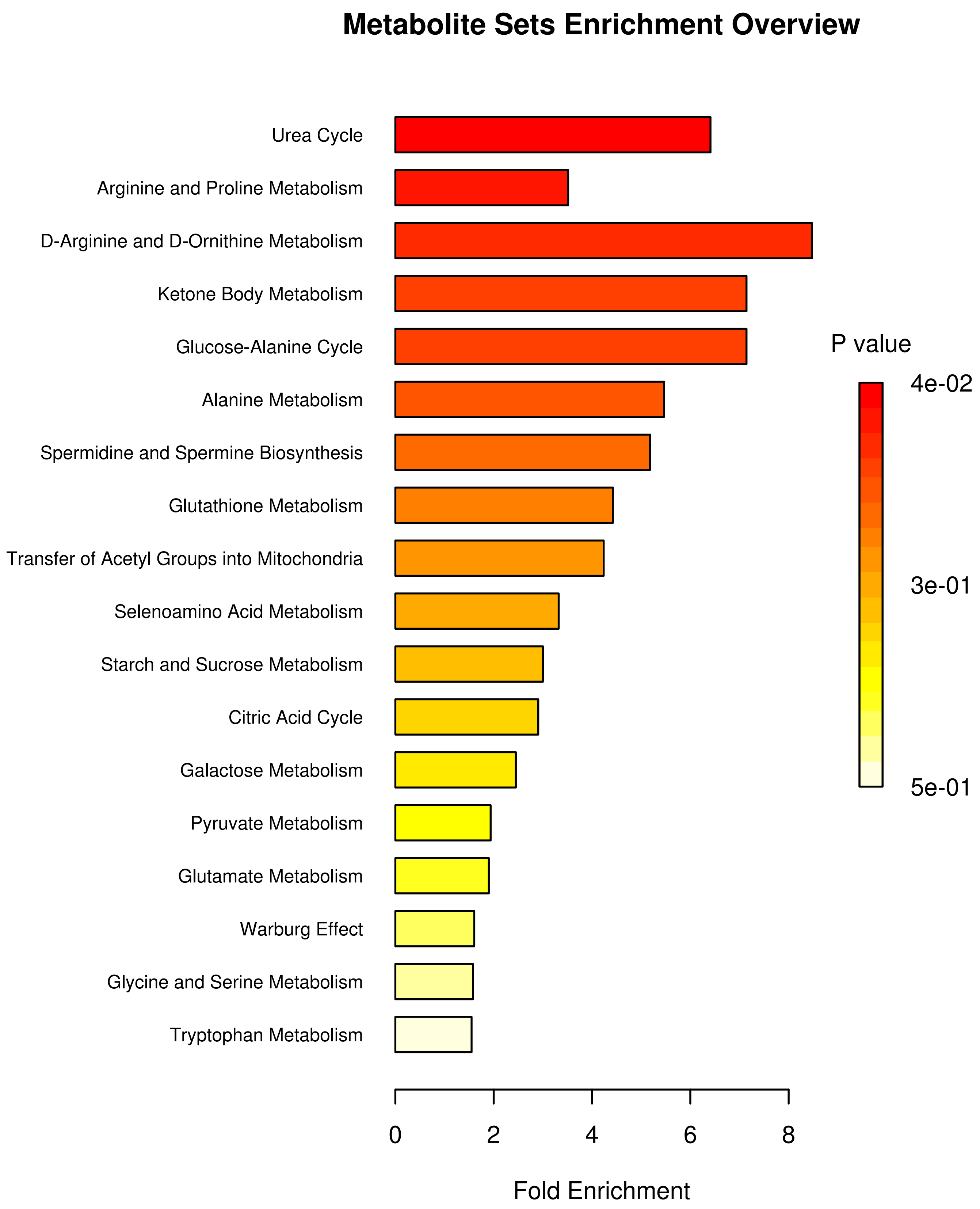
Metabolite Sets Enrichment Overview
Urea Cycle
Arginine and Proline Metabolism
D-Arginine and D-Ornithine Metabolism
Ketone Body Metabolism
Glucose-Alanine Cycle
Alanine Metabolism
Spermidine and Spermine Biosynthesis
Glutathione Metabolism
Transfer of Acetyl Groups into Mitochondria
Selenoamino Acid Metabolism
Starch and Sucrose Metabolism
Citric Acid Cycle
Galactose Metabolism
Pyruvate Metabolism
Glutamate Metabolism
Warburg Effect
Glycine and Serine Metabolism
Tryptophan Metabolism
P value
4e-02
3e-01
5e-01
0
2
4
6
8
Fold Enrichment
